# Supplementary figures and images for: Invariant recognition drives neural representations of action sequences
Source: PLoS Comput Biol. 2017 Dec 18;13(12):e1005859. doi: 10.1371/journal.pcbi.1005859 (PMC5749869; doi:10.1371/journal.pcbi.1005859)

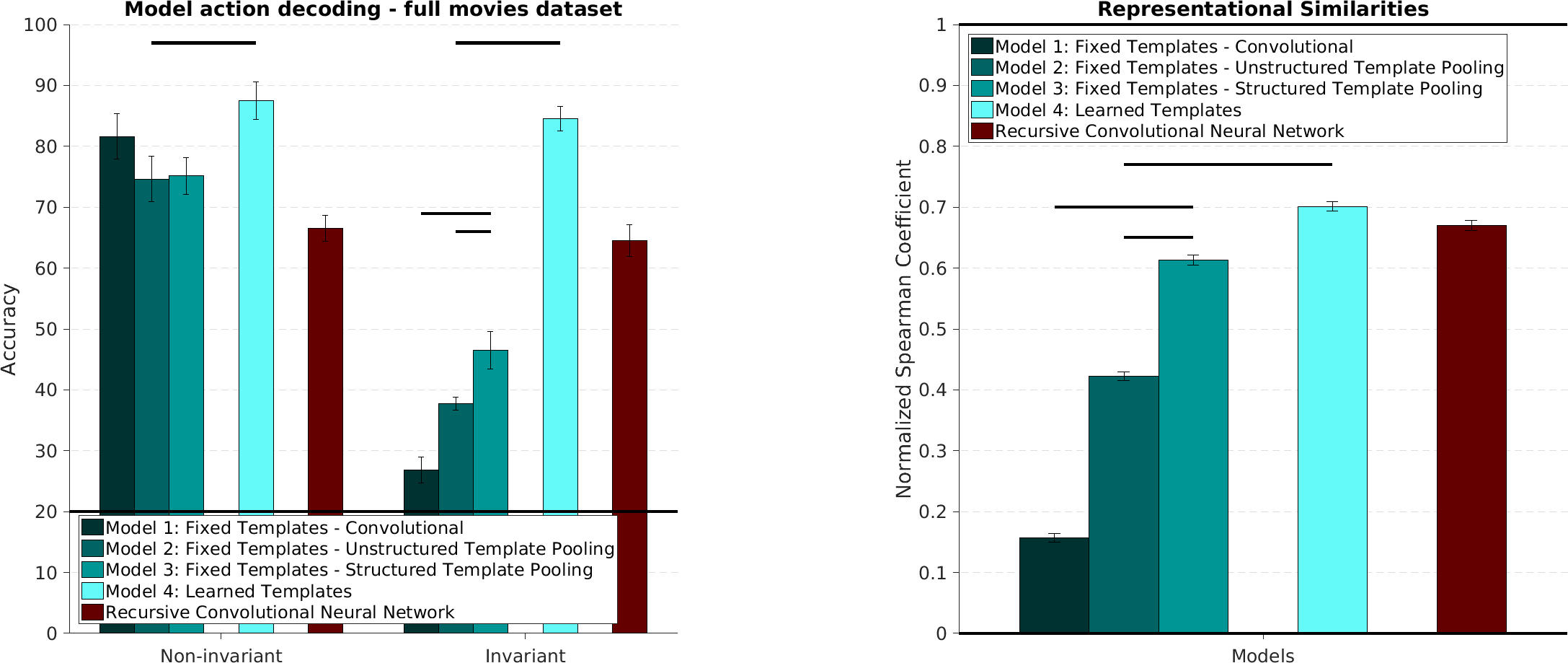

Supplement: S1 Fig — a) Classification accuracy, within and across changes in 3D viewpoint for a Recurrent Convolutional Neural Network. This architecture does not outperform a purely feedforward baseline. b) A Recurrent Convolutional Neural Network does not produce a dissimilarity structure that better agrees with the neural data than a purely feedforward baseline. (TIF) [file pcbi.1005859.s001.tif]

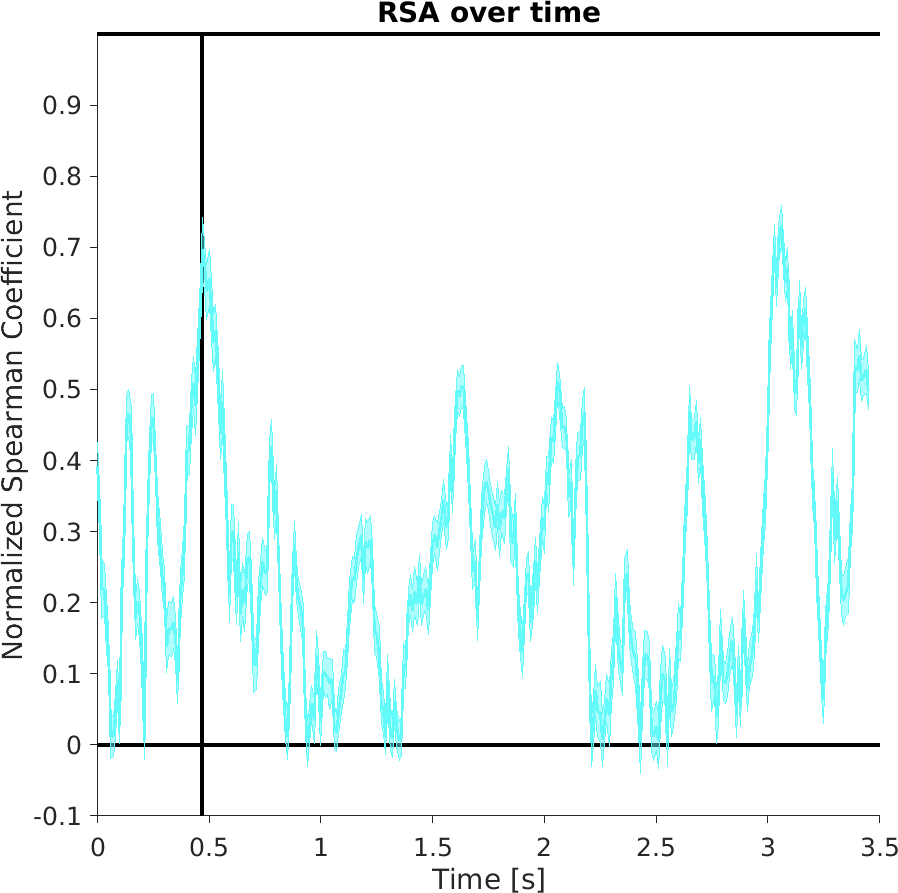

Supplement: S2 Fig — Neural data is most informative for action content of the stimulus at the time indicated by the vertical black line [33]. (TIF) [file pcbi.1005859.s002.tif]
