# Supplementary material for: Invariant recognition drives neural representations of action sequences
Source: PLoS Comput Biol. 2017 Dec 18;13(12):e1005859. doi: 10.1371/journal.pcbi.1005859 (PMC5749869; doi:10.1371/journal.pcbi.1005859)
Supplement: S1 Text — (DOCX) [file pcbi.1005859.s003.docx]

## Supplementary Text

## Recurrent Neural Networks

We constructed a Recurrent version of our hierarchical model and assessed how well the resulting representation supports invariant and non-invariant action recognition as well as how closely it matches neural data, in order to try and account for these feedback mechanisms. The network architecture is identical to the Model with Learned Templates used for Experiment 1, Experiment 2 and Experiment 3 (see Methods section). The Linear layers, however, were replaced by a Recurrent structure with a hidden state of 128 units, resulting in an architecture similar to the one described in [11]. Classification accuracy on an invariant and a non-invariant action recognition tasks as well as quality of match between the representation and neural data are presented in (S1 Fig). All procedures and data splits are identical to those described in the Methods section in the main text. Recurrent neural networks do not exceed the performance of simpler Feedforward models on the particular action recognition task presented in this work, however it is hard to draw definitive conclusions about their relevance to neural modeling, given the fact that the stimuli we used were relatively short and contained pre-segmented actions.

## RSA over time

For completeness, we report here the absolute value of the normalized Spearman Correlation Coefficient between the model with learned templates and the neural data. The time series is obtained by repeating the procedure outlined in the methods section using neural data from each time point (stimulus onset is at time 0) and time point used for figures in the main text corresponds to the vertical black line. Results are presented in (S2 Fig).
